# Reliability of Large Language Model-Based Chatbots Versus Clinicians as Sources of Information on Orthodontics: A Comparative Analysis

**DOI:** 10.3390/dj13080343

**Published:** 2025-07-24

**Authors:** Stefano Martina, Davide Cannatà, Teresa Paduano, Valentina Schettino, Francesco Giordano, Marzio Galdi

**Affiliations:** Department of Medicine, Surgery and Dentistry “Scuola Medica Salernitana”, University of Salerno, Via Allende, 84081 Baronissi, Italy

**Keywords:** orthodontics, malocclusion, artificial intelligence, chatbots, health information

## Abstract

**Objectives**: The present cross-sectional analysis aimed to investigate whether Large Language Model-based chatbots can be used as reliable sources of information in orthodontics by evaluating chatbot responses and comparing them to those of dental practitioners with different levels of knowledge. **Methods**: Eight true and false frequently asked orthodontic questions were submitted to five leading chatbots (ChatGPT-4, Claude-3-Opus, Gemini 2.0 Flash Experimental, Microsoft Copilot, and DeepSeek). The consistency of the answers given by chatbots at four different times was assessed using Cronbach’s α. Chi-squared test was used to compare chatbot responses with those given by two groups of clinicians, i.e., general dental practitioners (GDPs) and orthodontic specialists (Os) recruited in an online survey via social media, and differences were considered significant when *p* < 0.05. Additionally, chatbots were asked to provide a justification for their dichotomous responses using a chain-of-through prompting approach and rating the educational value according to the Global Quality Scale (GQS). **Results**: A high degree of consistency in answering was found for all analyzed chatbots (α > 0.80). When comparing chatbot answers with GDP and O ones, statistically significant differences were found for almost all the questions (*p* < 0.05). When evaluating the educational value of chatbot responses, DeepSeek achieved the highest GQS score (median 4.00; interquartile range 0.00), whereas CoPilot had the lowest one (median 2.00; interquartile range 2.00). **Conclusions**: Although chatbots yield somewhat useful information about orthodontics, they can provide misleading information when dealing with controversial topics.

## 1. Introduction

Online Health Information Seeking Behavior (OHISB) identifies a general behavioral pattern of using the Internet as a primary tool to search for health information [1]. As communication and information technologies improve, OHISB is increasingly becoming a global trend [2]. Indeed, searching for health information online has become a preferred method because of its accessibility, comprehensive coverage, ease of use, low cost, interactivity, and anonymity [3,4].

OHISB is thought to positively influence health information consumers by improving their knowledge of health-related topics, influencing opinion formation, and resulting in desirable behavior, as patients are more likely to adhere to treatment after receiving sufficient information about their health conditions [5,6]. However, the reliability and accuracy of health information sources on the Internet remain a major concern [7]. Notably, several investigations have found inaccurate and misleading health-related content on social media, which represents the most appealing source of medical and dental information for laypeople [8,9,10,11]. Such inaccurate information may negatively affect awareness and attitudes of patients towards health-related topics [12,13].

The introduction of Large Language Models (LLMs) has offered a new way for Internet users to access health information [14,15]. LLMs are artificial intelligence (AI) systems that are pre-trained on large volumes of text from datasets, books, articles, and web sources through various word prediction tasks [16]. Through further fine-tuning, including varying levels of human feedback, LLMs develop Natural Language Processing (NLP) capabilities and can generate relevant text responses in plain language to free-text prompts [17,18]. Early data suggests that these AI-powered chatbots can even provide empathetic responses to patients [19]. These chatbots are also able to address patients’ queries and provide answers in an easy-to-understand manner [17,20].

Patients have shown interest in seeking medical advice from chatbots [21], leading to a rapid increase in studies evaluating their ability to provide reliable health advice to both physicians and patients [22]. These investigations have focused on the ability of chatbots to synthesize evidence and provide accurate health guidance on topics such as screening, diagnosis, treatment, and disease prevention [23]. However, chatbots are generally not designed for medical use, and there is currently no regulatory oversight for their use in healthcare [24]. In addition, generative AI-powered chatbots pose unique risks, including the potential to confidently generate incorrect responses and disseminate misleading information and advice [25]. As a result, the aforementioned studies found low levels of chatbot performance, highlighting concerns about their limitations and associated risks [26].

Previous studies have shown that LLMs have promising potential as an aid in the implementation of evidence-based dentistry. However, models occasionally exhibit inaccuracies, outdated content, and a lack of source references. With regard to orthodontics, recent investigations found that AI-based chatbots can be useful for patient education and guidance [27,28]. However, these studies shared the limitations of categorizing the responses of chatbots as true and false, whereas the quality of chatbots’ detailed explanations was not evaluated.

Therefore, the aim of the present study was to assess the accuracy and consistency of AI-based chatbots as a source of information on orthodontics by comparing the answers they provided to orthodontic questions with those given by general dental practitioners (GDPs) and orthodontic specialists (Os) and by rating the educational value of their detailed explanations.

## 2. Materials and Methods

### 2.1. Study Design

The present study was developed following the Strengthening the Reporting of Observational Studies in Epidemiology (STROBE) guidelines [29] and adhering to ethical guidelines for research involving human participants in the use of AI in dentistry. Ethical approval was waived for this study since no patient data was used and clinicians voluntarily self-enrolled.

Data collection was carried out between March 2024 and January 2025 from two different sources:

1. Artificial Intelligence-based chatbots, including ChatGPT-4, Claude-3-Opus, Gemini 2.0 Flash Experimental, Microsoft Copilot, and DeepSeek 1.0.0.
2. Clinicians, including general practitioners and orthodontists (i.e., practitioners who have obtained a specialty in orthodontics).

To generate a set of frequently asked orthodontic questions, the Google Trends website (freely available at https://trends.google.com/trends/, accessed on 24 February 2024) was searched to determine the most frequently used search term related to orthodontics worldwide in the past five years on Google. The term with the highest search volume was “malocclusion”. Accordingly, the most frequently searched queries associated with “malocclusion” were identified through Google (“Frequently Asked Questions about Malocclusion”) and chatbots (“What are 10 frequently asked questions with the word “malocclusion”?). Two orthodontic specialists (S.M. and M.G.) eliminated duplicates and unscientific inquiries and selected only dichotomous questions. Identified questions were clarified if needed, and an 8-item yes/no questionnaire was created (Table 1).

### 2.2. Survey Dissemination

The 8-item yes/no questionnaire was released as an online survey created using Google Forms (http://www.google.com/forms/about/, accessed on 27 February 2024). It was distributed through a standardized recruitment email that included a link to the survey and a cover letter explaining the aim of the study, assuring participants of the anonymity of their responses, and requesting participation. There was no financial incentive to participate in the survey.

Clinicians were recruited via Facebook (Meta Platforms), as this has proven to be a cost-effective means of recruiting for online survey research.

The electronic message was disseminated through various modalities supported by the Facebook platform, including public posts on dental network group pages and private messages.

Participants could only respond once to the survey, and it was not possible to change responses after successful submission. It was made clear to all participants that participation was entirely voluntary, and that they could choose to stop filling out the questionnaire at any time and for any reason, without facing any penalties.

At the beginning of the survey, participants were asked if they were orthodontists (i.e., with a specialty in orthodontics) or general dentists in order to enable a comparison of answers across the two categories, as well as to determine how long they had been in the dental profession.

The target sample size was estimated using R. Software, version 4.1.0 (May 2021, R Foundation for Statistical Computing, Vienna, Austria), with Cochran’s sample size formula for prevalence studies [30]. Since the total number of dental practitioners in Italy is approximately 60,000 according to Eurostat data (https://ec.europa.eu/eurostat/data/database; accessed on 27 February 2024), the target sample size for the survey was estimated to be 382 participants, at a 95% confidence level and 3% margin of error.

The initial dissemination of the standardized recruitment message and survey link occurred on the Facebook platform on 3 March 2024. The first messages were sent on the same day, and follow-up reminders (messages and posts) were sent at one, two, and four weeks.

### 2.3. Chatbot Analysis Process

A standardized prompt-template using a chain-of-thought prompting approach was developed and used for all LLM questions [31,32]. The prompts used are detailed in Table 2.

To evaluate the consistency of the chatbots, two users experienced in chatbot use (D.C. and V.S.) asked each question in four different attempts, each time starting a new conversation after clearing the browser history and cache. Moreover, the questions were resubmitted to the chatbots from different computer systems, with a cleared history and cache, after three, six, and nine days, for a total of 20 attempts. Chatbots were set as follows: memory and model improvement disabled; no preference in customization.

The same users then asked the chatbots the same questions again, requiring them to justify their answers, and rated the chatbots’ responses educational value according to the five-point Global Quality Scale (GQS) criteria [33,34]:

- Score 1: Poor quality, very unlikely to be of any use to patients.
- Score 2: Poor quality, but some information present; of very limited use to patients.
- Score 3: Suboptimal flow, some information covered but important topics missing; somewhat useful to patients.
- Score 4: Good quality and flow, most important topics covered; useful to patients.
- Score 5: Excellent quality and flow; highly useful to patients.

To address potential sources of bias, the responses of chatbots were evaluated independently and blindly by two certified orthodontic dentists (S.M. and M.G.) against the generated list of answers. Any discrepancies were resolved through consensus.

### 2.4. Data Collection and Statistical Analysis

Data collected from the questionnaire and the responses of the chatbot were qualitatively synthesized by means of descriptive statistics analysis using Microsoft Excel software 2019 (Microsoft Corporation, Redmond, WA, USA), and frequencies and percentages were calculated for each item.

Cronbach’s α was calculated to assess chatbot consistency in answering questions, with a threshold value of 0.80 indicating excellent reliability.

Chi-squared test or Fisher’s exact test, depending on the observed frequencies, was performed to assess differences in the answers given by Os and GPs to different items of the questionnaire, as well as differences in the responses of dentists and chatbots to the various questionnaire items.

To assess the quality of the responses provided by chatbots, the median and interquartile range (IQR) were calculated for the GQS values assigned to responses given by individual chatbots to all questions and to individual questions for all chatbots.

Statistical analysis was carried out using the IBM SPSS Statistics 29.0.0 software (IBM Corporation, Armonk, NY, USA). A significance level of *p* < 0.05 was considered statistically significant.

## 3. Results

### 3.1. Dentists’ Responses to the Most Frequently Internet Searched Queries Associated with “Malocclusion”

A total of 458 dentists participated in the present study, notably 284 GDPs (62.0%), and 174 Os (38.0%). GDPs and Os were similar in terms of years in the dental profession (less than 10 years: 146 GDPs, 96 Os, *p* = 0.434; 10–20 years: 92 GDPs, 54 Os, *p* = 0.762; more than 20 years: 46 GDPs, 24 Os, *p* = 0.488). Between groups, differences were found in the responses to the questions Q3 and Q4, suggesting that, on average, GDPs were more likely to support the relationships between malocclusion and temporomandibular disorder (TMD) signs and symptoms, as well as malocclusion and posture, compared to Os, as shown in Table 3. Indeed, 61.2% of GDPs (174) believed that malocclusion can affect posture, whereas only 46.5 % of Os (81) believed the same (*p* = 0.002). Similarly, most GDPs (54,9%) considered malocclusion a risk factor for the onset of TMDs, while only 25.9% of Os agreed with this statement (*p* < 0.001). No other differences were found between the two groups’ answers.

### 3.2. Chatbot Analysis Results

The analysis of Cronbach’s α revealed a high degree of consistency in chatbot responses, ranging from 0.93 to 0.99.

When compared with GDPs’ and Os’ ones, all chatbots’ responses to the questionnaire were found to be statistically different, except for Q6, as shown in Table 4 and Table 5.

**Figure 1:**
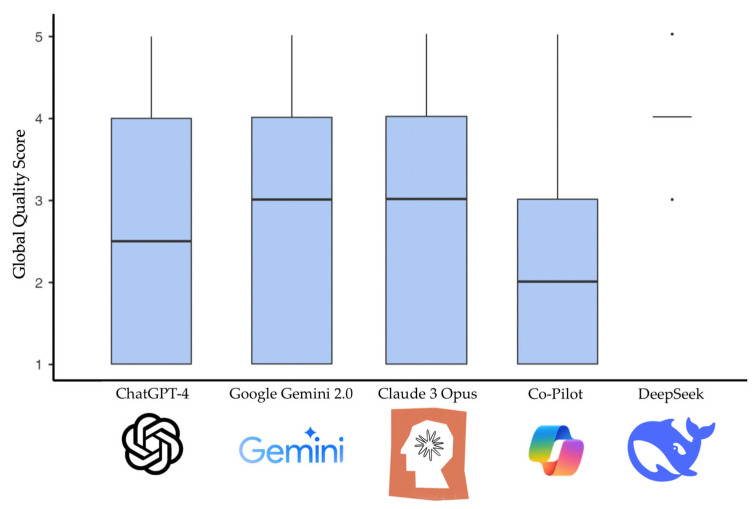
Distribution of the GQS scores among the different chatbots.

When evaluating the chatbots using the GQS, DeepSeek achieved the highest median score (median 4.00; IQR 0.00), whereas CoPilot had the lowest median (median 2.00; IQR 2.00). Figure 1 shows the distribution of the GQS scores among the different chatbots.

**Figure 2:**
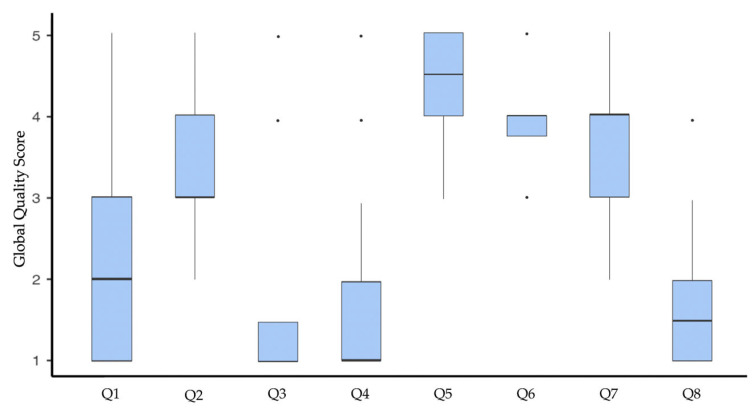
Distribution of the GQS scores among the different questions.

Regarding the GQS evaluation of individual questions, the highest-quality responses were observed for Q6 (median 4.00; IQR 0.25). In contrast, the lowest performance was associated with Q3 and Q4 (median 1.00; IQR 1.00). Figure 2 shows the distribution of the GQS scores for the different questions.

## 4. Discussion

The present cross-sectional study aimed to compare the performance of several prominent AI chatbots—namely ChatGPT-4, Claude-3-Opus, Gemini 2.0 Flash Experimental, Microsoft Copilot, and DeepSeek—assessing their reliability and consistency in answering a set of orthodontic questions and comparing them with that of general dental practitioners and orthodontists.

The analysis of the responses to the questionnaire found that both Os and GDPs did not support the causal role of the lower third molar in the onset of malocclusion (Table 2), in accordance with recent investigations on the topic [35]. Instead, the chatbots provided answers that support the potential of a third molar eruption in determining malocclusion, notably anterior dental crowding (Table 3 and Table 4). Actually, the effect of mandibular third molars on crowding of mandibular teeth remains an ongoing topic of discussion among researchers and clinicians. Indeed, some studies found a significant association between impacted third molars with insufficient mesiodistal distance and dental crowding, resulting from the force applied by the molars to other teeth [36]. However, evidence supporting the contribution of lower third molars to mandibular crowding and lower arch constriction is weak, marred by the absence of randomized studies and the presence of uncontrollable confounding factors, such as growth-related changes, muscular factors, periodontal ligament traction, bone adaptation, masticatory force, dental agenesis, and previous dental extractions in the studied populations. Additionally, the low methodological quality of the existing studies further weakens the evidence [37]. The information provided by chatbots in supporting the role of third molars in the pathogenesis of malocclusions may influence the patients’ perception of the scientifically unsupported need for asymptomatic molars extraction to prevent dental crowding, which could be misleading. Overall, in relation to the current literature, the quality of information provided by chatbots to justify their response to Q1 was considered poor.

The possibility of malocclusions affecting chewing, speech patterns, and swallowing was supported by Os, GPDs, and chatbots (Table 1, Table 3 and Table 4). On this topic, there is a general consensus in the literature that certain malocclusions, such as an open bite, may affect the pattern of phonation and swallowing [38], and, similarly, severe malocclusions are potentially capable of altering muscle function and thus chewing [39]. However, the questionnaire presented in this study did not distinguish between different types of malocclusions, and thus clinicians’ responses that deviate from the majority must be interpreted given the impossibility of generalizing the association between malocclusion and functional problems. The strength of the evidence in the literature regarding these topics was also reflected in the ability of chatbots to provide adequate arguments to justify their responses, resulting in good quality, useful information for Internet users, according to the findings of the GQS analysis (Q4: median 4.50, IQR 1.00; Q2: median 3.00, IQR 1.00; Figure 2).

An agreement between dental practitioners and chatbots was also found in recognizing heritability among the factors contributing to the determination of malocclusion, as well as in acknowledging the potential for untreated malocclusion to worsen over time (Table 1, Table 2 and Table 3). This agreement stems from the consensus in the literature on the genetic component underlying malocclusions [40] and on the possibility of dentofacial deformities to worsen with growth [41], which is reflected in the good-quality information provided by the chatbots (Q6: median 4.00; IQR 0.25; Q7: median 4.00; IQR 1.00; Figure 2).

The association between occlusion and posture was denied by Os, whereas general practitioners indicated a possible link (Table 3), which is also supported by chatbot responses (Table 4 and Table 5). This could be explained by the ambiguity of the scientific literature related to this topic. Indeed, although some recent reviews reported evidence of a possible association between sagittal malocclusion and head and body posture [42,43], primitive study design, methodological heterogeneity, small sample sizes, and definition and analysis of outcomes severely limit the generalizability of these results. Coherently, the only existing meta-analysis on the topic found no sufficiently robust evidence supporting an association between head and neck posture with sagittal malocclusion [44]. Despite the ambiguity of the relevant literature, chatbots support the link between occlusion and posture, though with poor-quality, low evidence-based information (Q3: GQS median 1.00, IQR 1.00; Figure 2), potentially leading to inappropriate management of both postural problems and malocclusions. Furthermore, it is crucial for patients to seek consultation with a specialist rather than a GDP, as this can potentially result in inappropriate management of the underlying postural issue through orthodontic interventions.

Similarly, chatbots claimed a relationship between malocclusion and TMD signs and symptoms (Table 3 and Table 5). General dental practitioners also supported the etiopathogenetic role of malocclusion in TMD development or progression, whereas specialists did not (Table 3). For instance, a recent web-based analysis on TMD-related content showed that the existing online information ascribes TMD to occlusal problems, including malocclusion, and recommends addressing these problems to manage TMD [45,46]. Although a recent meta-analysis suggested a higher prevalence of TMD in subjects with malocclusion, notably Class II and posterior unilateral crossbite [47], the lack of standardized TMD diagnostic criteria, malocclusion classifications, and representative sampling methods across available studies makes it impossible to establish a clear correlation or to know if and when a malocclusion may unbalance the stomatognathic system and cause TMD signs and symptoms. After all, the role of occlusion in the TMD has been downplayed in favor of cognitive, emotional, and behavioral factors [48,49]. Therefore, the literature does not support orthodontic treatment as a therapy for TMD management and suggests routinely performing TMD-related examinations before and throughout the duration of orthodontic treatment in order to start treatment only in patients without pain and to temporarily discontinue treatment if pain occurs [50]. However, the inaccurate information provided by chatbots (Q4: GQS median 1.00, IQR 1.00; Figure 2) may influence patients’ attitudes, leading them to seek orthodontic treatment to address their pain or dysfunctional symptoms. Since the level of knowledge on this topic is low even among clinicians, especially general dentists, there is a risk of going against proper TMD management.

Lastly, the ability of malocclusion to result in the onset of headaches is supported by both clinicians and chatbots (Table 2, Table 3 and Table 4). Although a recent systematic review suggested that certain malocclusions, notably Class II malocclusion, might be associated with a higher headache prevalence [51], the level of evidence is very low. Moreover, due to the heterogeneity of headaches described in the most recent International Classification of Headache Disorders (ICHD-3) [52], it is not possible to formulate a definitive answer to this question. In fact, most studies on this topic either do not specify the type of headache or assume that it is a headache attributable to TMD. In addition, it is unclear whether the headache is a direct consequence of the malocclusion, resulting from adaptations of the stomatognathic system to the occlusal imbalances that might stimulate the trigeminal nerve, potentially leading to central sensitization [53], or rather the result of the malocclusion’s impact on the patient’s quality of life [54]. The information provided by chatbots on this topic was misleading. However, DeepSeek exhibited a noticeably higher GQS than other chatbots since it mentioned the possible implication of the psychosocial impact of malocclusions.

Based on the above, the accuracy of chatbots as public sources of information on orthodontics appeared to be influenced by the level of evidence available in the literature relative to the topic analyzed since the greater the consensus in the literature, the more accurate the answer provided proved to be. Indeed, it must be considered that chatbots answer questions by integrating information from external sources such as databases, scientific articles, and websites. When there is a high degree of agreement among these sources, the resulting answer will have greater accuracy, so much so that they can be equated with those of clinicians, as also shown by previous studies [55]. Instead, when there is no agreement on a topic, AI-based chatbots were prone to generate plausible-sounding but inaccurate content [24].

The significant differences seen between the chatbot and clinician responses for most questions, except for heritability, matched the findings of Metin and Goymen [27]. Their research showed that AI-driven chatbots had higher accuracy compared to dental students and general dentists but generally performed worse than orthodontists. Salmanpour et al. confirmed this claim, demonstrating that expert responses are consistently better than those of chatbots in complex areas of orthodontics [28]. 

However, Zhou et al. suggested that ChatGPT-4 might provide more accurate answers than specialists when handling common questions about orthodontics [56]. Similarly, Hassan et al. found that Google Gemini reached 100% accuracy in answering questions about TMD [57]. These contradictions indicate that generalizing the performance of AI is not possible, depending on many factors such as the specific model, the type of question, and the evaluation criteria.

Differences in accuracy observed among the different chatbots highlighted that not all LLMs are equally proficient in handling specialized medical or dental information. Differences in their training datasets, underlying model architectures, and the specific fine-tuning techniques employed likely contribute to these variations in their ability to accurately and comprehensively address questions related to malocclusion. DeepSeek’s superior performance could potentially be attributed to specific characteristics of its training data or model architecture that make it particularly well-suited for processing and responding to the types of questions included in this study. A key strength of DeepThink was its open-source nature, which allowed users to access and modify the source code based on their needs. This learning process used data from public sources, enabling the model to update itself continuously with the latest scientific and medical findings. As a result, this approach helped it keep up with new trends in health care and made it more effective over time [58]. This noted diversity in performance exists throughout literature. For example, Dermata et al. found that ChatGPT-4 ranked highest in pediatric dentistry among six LLMs, closely followed by Gemini Advanced and ChatGPT-4o [59]. In addition, Özbay et al. further found that ChatGPT-4 outperformed ChatGPT-3.5 and Google Bard in endodontics [60]. Hassan et al. found that ChatGPT-4o was much more complete and more accurate than other models for TMD questions [57]. On the other hand, Makrygiannakis et al. reported that the best performance in orthodontics came from Microsoft Bing Chat and was followed by ChatGPT-4 [61]. This finding indicates that the "best" chatbot is dependent upon the specific area and evaluation method.

Interestingly, the results of the present study found excellent inter-chatbot reliability. When the same chatbot was asked the same question several times, by different users and at different times, the answer was always the same, and the quality of the related argument was almost always consistent. This finding was in contrast with those of previous studies, which reported that chatbots may generate different responses to the same prompt if asked multiple times or asked by different users or at different times.

This study offers several notable strengths. The study simultaneously evaluates four different LLMs, which is a rare approach, as most existing research focuses on a single model—typically ChatGPT, which was the first to be publicly available and remains the most widely known. Another strength of this study is that it includes not only quantitative findings but also qualitative results, such as evaluators’ detailed comments, which provide deeper insights into the LLMs’ performance and reveal some of their limitations.

The main limitation of the study relates to the content of the questions asked of the chatbots. Since the questions were identified based on Internet search trends, it was not possible to establish a consensus “gold standard” against which to compare the artificial intelligence responses, and thus experts had to rely solely on their own subjective judgment to assess the correctness and completeness of the answers. However, as the same study highlighted, experts also disagree on some of the issues analyzed. Nevertheless, considering the purpose of the study, selecting frequently asked questions provided a more accurate representation of the actual usefulness of chatbots as a source of information for Internet users. However, it should be considered that it was impossible to know who conducted the searches through the identified search terms—doctors, students, or patients—limiting the ability to contextualize and correctly interpret data. Secondarily, a potential selection bias of survey participants should be considered, since they were geographically homogeneous, limiting the generalizability of the results. Another major limitation concerns the operators’ judgment of the quality of chatbot responses, whose inadequate or incomplete content may have been camouflaged through fluid and articulate language, according to the concept of hallucination of LLMs. Another selection bias concerns the choice of questions arbitrarily conducted by operators, including questions the answer to which may be contained in the gray literature that is not consulted by chatbots. Lastly, it is important to highlight that the responses reflect the performance of the LLMs at the time the research was conducted, and this may evolve over time, which is an inherent limitation of studies focused on technological advancements.

## 5. Conclusions

The results of the present study found that AI-based chatbots provide information about orthodontics with excellent reproducibility. According to GQS, this information has a medium level of accuracy, with DeepSeek showing the statistically significant highest performance and proving to be somewhat useful to patients. However, when dealing with controversial topics, the accuracy of chatbots was weak compared to clinicians’ opinions on the same topics.

## Figures and Tables

**Table 1:** Eight-item questionnaire developed on the basis of most frequently Internet-searched queries associated with “malocclusion”.

Questions
Q1: Can the third molar eruption cause malocclusion?
Q2: Can malocclusion affect chewing? Q3: Can malocclusion affect posture? Q4: Can malocclusion cause TMJ sound and/or jaw pain? Q5: Can malocclusion affect speech or swallowing? Q6: Can malocclusion be hereditary? Q7: Can untreated malocclusion worsen over time? Q8: Can malocclusion cause headache?

**Table 2:** Standardized prompts using chain-of-thought prompting approach.

Prompt	
P1	Step 1: Can the third molar eruption cause malocclusion? Give me a dichotomous answer. Step 2: Can you support your answer with reasons?
P2	Step 1: Can malocclusion affect chewing? Give me a dichotomous answer. Step 2: Can you support your answer with reasons?
P3	Step 1: Can malocclusion affect posture? Give me a dichotomous answer. Step 2: Can you support your answer with reasons?
P4	Step 1: Can malocclusion cause TMJ sound and/or jaw pain? Give me a dichotomous answer. Step 2: Can you support your answer with reasons?
P5	Step 1: Can malocclusion affect speech or swallowing? Give me a dichotomous answer. Step 2: Can you support your answer with reasons?
P6	Step 1: Can malocclusion be hereditary? Give me a dichotomous answer. Step 2: Can you support your answer with reasons?
P7	Step 1: Can untreated malocclusion worsen over time? Give me a dichotomous answer. Step 2: Can you support your answer with reasons?
P8	Step 1: Can malocclusion cause headache? Give me a dichotomous answer. Step 2: Can you support your answer with reasons?

**Table 3:** Responses to the most frequently Internet searched queries associated with “malocclusion” of general dental practitioners (GDP) and orthodontists (O). Between-group differences were measured with the Chi-squared test.

Questions	Response	Total	GDP	O	*p*-Value
Q1: Can the third molar eruption cause malocclusion?	Yes	125	82	43	0.332
	No	333	202	131	
Q2: Can malocclusion affect chewing?	Yes	332	208	124	0.646
	No	126	76	50	
Q3: Can malocclusion affect posture?	Yes	255	174	81	0.002 *
	No	203	110	93	
Q4: Can malocclusion cause TMJ sound and/or jaw pain?	Yes	201	156	45	<0.001 *
	No	257	128	129	
Q5: Can malocclusion affect speech or swallowing?	Yes	273	178	95	0.087
	No	185	106	79	
Q6: Can malocclusion be hereditary?	Yes	447	276	171	0.458
	No	11	8	3	
Q7: Can untreated malocclusion worsen over time?	Yes	407	256	151	0.267
	No	51	28	23	
Q8: Can malocclusion cause headache?	Yes	298	182	116	0.574
	No	160	102	58	

* *p* < 0.05, significant association.

**Table 4:** Responses to the most frequently Internet searched queries associated with “malocclusion” of general dental practitioners (GDPs) and chatbots (Cs). Between-group differences were measured with Fisher’s exact test.

	Response	GDP	C	*p*-Value
Q1: Can the third molar eruption cause malocclusion?	Yes	82	86	<0.001 *
	No	202	14	
Q2: Can malocclusion affect chewing?	Yes	208	100	<0.001 *
	No	76	0	
Q3: Can malocclusion affect posture?	Yes	174	100	<0.001 *
	No	110	0	
Q4: Can malocclusion cause TMJ sound and/or jaw pain?	Yes	156	100	<0.001 *
	No	128	0	
Q5: Can malocclusion affect speech or swallowing?	Yes	178	100	<0.001 *
	No	106	0	
Q6: Can malocclusion be hereditary?	Yes	276	100	0.090
	No	8	0	
Q7: Can untreated malocclusion worsen over time?	Yes	256	100	<0.001 *
	No	28	0	
Q8: Can malocclusion cause headache?	Yes	182	100	<0.001 *
	No	102	0	

* *p* < 0.05, significant association.

**Table 5:** Responses to the most frequently Internet searched queries associated with “malocclusion” of orthodontists (Os) and chatbots (Cs). Between-group differences were measured with Fisher’s exact test.

	Response	O	C	*p*-Value
Q1: Can the third molar eruption cause malocclusion?	Yes	43	86	<0.001 *
	No	131	14	
Q2: Can malocclusion affect chewing?	Yes	124	100	<0.001 *
	No	50	0	
Q3: Can malocclusion affect posture?	Yes	81	100	<0.001 *
	No	93	0	
Q4: Can malocclusion cause TMJ sound and/or jaw pain?	Yes	45	100	<0.001 *
	No	129	0	
Q5: Can malocclusion affect speech or swallowing?	Yes	95	100	<0.001 *
	No	79	0	
Q6: Can malocclusion be hereditary?	Yes	171	100	0.187
	No	3	0	
Q7: Can untreated malocclusion worsen over time?	Yes	151	100	<0.001 *
	No	23	0	
Q8: Can malocclusion cause headache?	Yes	116	100	<0.001 *
	No	58	0	

* *p* < 0.05, significant association.

## Data Availability

The datasets used and analyzed during the current study are available from the corresponding author upon reasonable request.

## Abbreviations

The following abbreviations are used in this manuscript:

AI	Artificial Intelligence
C	Chatbot
GQS	Global Quality Score
ICHD-3	International Classification of Headache Disorders
IQR	Interquartile Range
LLM	Large Language Model
NLP	Natural Language Processing
O	Orthodontic specialists
OHISB	Online Health Information Seeking Behavior
Q	Question
STROBE	Strengthening the Reporting of Observational Studies in Epidemiology
TMD	Temporomandibular Disorder
